# COFACTOR-residential: Hourly electricity and heating data from residential buildings in Norway

**DOI:** 10.1016/j.dib.2026.112580

**Published:** 2026-02-12

**Authors:** Åse Lekang Sørensen, Synne Krekling Lien, Harald Taxt Walnum

**Affiliations:** Sintef Community Pb 124 Blindern, 0314 Oslo, Norway

**Keywords:** Time series data, Energy use, Electricity, Space heating, Domestic hot water, Apartment buildings, Single-family houses, Cabins

## Abstract

This data descriptor describes three datasets with hourly residential energy use data and building information data from apartments, cabins and single-family houses. The first dataset contains 29 files with energy use data from 29 apartment block condominiums, with hourly time series of both electricity and delivered heat. The second dataset includes 20 files from Risvollan borettslag in Trondheim, covering heating and electricity use at the level of entire apartment blocks and heating centrals, as well as sub-metered electricity data from 1058 individual apartments. The third dataset consists of 194 files with electricity smart meter data from single-family houses, cabins, and apartments equipped with Sikom home energy management systems. The energy use data have been collected in co-operation with distribution system operators and utility providers, directly from building owners as well as from Sikom with permission from their users. Information about the Sikom users have been collected through a user survey. Each dataset is organized into human readable comma-separated txt files with a common structure that includes building information data, hourly records of electricity and heating energy use, and weather variables, with time series durations from several months up to multiple years per building. The data are suitable for tasks such as residential building energy analysis, load profile generation, energy disaggregation, classification tasks, forecasting of energy use, demand flexibility analysis, energy system analysis and other modelling tasks.

Specifications Table

**Table:** 

Subject	Engineering & Materials science
Specific subject area	Hourly energy use measurements and building information data from residential buildings (single family houses, apartments, apartment blocks and cabins).
Type of data	*Table; Processed human readable comma-separated (;) .txt files; readme (.txt). Decimal separator: “.”.* **Dataset 1:** 29 human readable comma-separated .txt files. Hourly energy use data (heating and electricity) and building information data for condominiums of apartment blocks connected to 29 different heating centrals, with one file per heating central. **Dataset 2:** 20 human readable comma-separated .txt files. Hourly energy use data (heating and electricity) and building information data for full apartment blocks and heating centrals as well as individual apartment electricity meters from 1058 apartments in Risvollan borettslag, a large condominium in Trondheim, Norway. **Dataset 3:** 194 human readable comma-separated .txt files. Hourly electricity use data and building information data from 194 individual single-family houses, cabins and apartments collected through their Sikom home energy management systems.
Data collection	**Dataset 1:** Data was collected through 3rd parties. Data from main electricity meters (AMS) were collected from the local distribution system operator (DSO), Elvia AS. Data from energy meters in district heating substations were collected from the local district heating company, Hafslund Celcio AS. Heat energy meters for DHW energy use were collected through the energy management system of the technical contractor delivering the system. Additional information about the buildings and their energy systems was collected through communication with the building owners and from public registers. **Dataset 2**: Information about the energy system and buildings was collected from the housing cooperative. Data from main electricity meters (AMS) were collected from the local DSO, Tensio AS. Data from heating meters were collected through the energy management system of the housing cooperative. Information about number of residents and age groups was collected from the national registry in Norway. **Dataset 3:** Data collected from user surveys and energy management system data from individual units (cabins, apartments and single-family houses) from Sikom Connect AS, and their equipment, from several locations in Norway. Data was cleaned through outlier detection and exclusion of buildings with not enough data. **General:** Weather data has been downloaded for the locations from MET Norway*.*
Data source location	**Dataset 1:** Institution: Apartment blocks City/Town/Region: Oslo and Bærum Country: Norway Explanation: Location given at sub-municipal level to preserve building anonymity. **Dataset 2:** Institution: Apartment blocks/apartments from Risvollan Borettslag City/Town/Region: Trondheim Country: Norway Latitude and longitude: 59° 58′ 13.7″ North, 10° 53′ 50.0″ East **Dataset 3:** Institution: Individual cabins, apartments and single-family houses City/Town/Region: Several locations. Country: Norway Explanation: Location given at municipal level to preserve user anonymity.
Data accessibility	Repository name: data.sintef.no / COFACTOR DOI Dataset 1: https://doi.org/10.60609/3ab7-ez93 DOI Dataset 2: https://doi.org/10.60609/qhpe-2e71 DOI Dataset 3: https://doi.org/10.60609/yd3z-e630 Direct URL to data: https://data.sintef.no/product/dp-b750d103-a434–45c2-ba5e-1af13f0a866f
Related research article	S. K. Lien, H. T. Walnum, and I. Sartori, ‘Estimating heating energy for domestic hot water from total heating measurements in apartment buildings: Comparison of methods for disaggregation and daily load profile estimation in a Norwegian case study.’, Submitted to Energy, 2025. [1]

## 1 Value of the Data

• This open data product consists of three different datasets with a total of 243 txt-files with hourly time series weather and energy use data from 2800 residential units with a total of approximately 250 000 m^2^ of heated floor area from Norway from both cabins, single-family houses, apartments and apartment blocks. The time series data have hourly resolution and a duration up to 4 years of data per building collected through a thorough approach in co-operation with building owners, energy providers and Sikom. The energy use data constitute novel primary data that have not previously been published and comprise both sub-metered energy use from residential buildings and smart meter data from Norwegian cabins, a data type that is currently lacking in the literature. The dataset can improve the knowledge on how residential buildings use energy. The dataset contains open, structured, and documented time series data compatible with common analysis tools (Python/R/Excel).
• The datasets can be used by law makers, grid operators, regulators and energy planners in Norway and the Nordic countries to generate yearly and typical daily load profiles for different types of residential buildings with uncertainty and weather dependencies. These load profiles can be used to evaluate current methods and normative values in building simulation methods, such as in the standard NS3031 supplied by Standards Norway. Such load profiles can also be used in scenario analysis of how changes in the climate, the composition of buildings in an area, the energy efficiency of buildings and/or the heating type of buildings would affect the future energy use, electricity demand and peak load of an area. In addition, dataset 2 contains sub-meters for 1058 single apartments from apartment buildings in the same area which can be used to investigate different methods for the calculation of the coincidence factor and the diversity factor of apartments.
• Researchers, PhD candidates, and students in the field of energy and building research can use this data to train, test, and validate data-driven models for heat load disaggregation in buildings with district or electric heating. Disaggregation, or non-intrusive load monitoring (NILM), separates a building’s total energy use into end-use components without additional sub-meters. Dataset 1 and Dataset 2 from this product were used in [1] to explore DHW energy disaggregation and investigate the best methods to generate typical load profiles for DHW, and the published datasets can be used as a benchmark to explore more methods for this task. Validated disaggregation models can reveal how individual loads and technologies contribute to total energy use and peak demand in buildings while typical load profiles can improve dimensioning of DHW systems in buildings, as well as be used for energy area planning.
• The datasets can provide insights on how residential buildings contribute to peak loads in the grid and their demand-side flexibility potential, which can be beneficial to researchers, grid-planners, DSOs and energy agencies. Dataset 2 was used in several research articles on these topics including evaluation of the heating system [2], electricity use [3], simulation of photovoltaic (PV) electricity generation [4], building renovation [5], domestic hot water (DHW) use [6], Electric Vehicle (EV) charging [7,8,9], and energy flexibility potential [10,11]. The current dataset can be used to replicate and validate these studies and perform alternative analysis on the peak loads of buildings and their sub-metered loads, as they contain sub-metered data for heating, domestic hot water heating, space heating, hot water heaters and EV-charging.
• In addition to energy use data, the datasets contain building information data (meta data) on each building or unit, including building category (apartment, apartment block, single-family house), heating system, size, and number of occupants. The datasets can be used to train classification algorithms to identify building characteristics when meta data is unavailable, reducing manual effort in future research data collection. The building information data can also be used to analyse how these parameters affect the hourly and yearly energy use of buildings which can be of interest to researchers in the field of building energy efficiency and energy sufficiency, as well as DSOs, energy planners and public agents, such as the Norwegian Water Resources and Energy Directorate.

## 2 Background

Detailed energy use data from buildings are essential for understanding energy use patterns, improving energy efficiency, reducing peak loads and for long term planning of the energy system. With sub-metered energy and labelled energy use data from residential buildings it is possible to explore how different loads contribute to the total heating load in residential buildings [1], as well as how residential buildings use energy and how they can contribute with demand side-flexibility [10,11]. This data descriptor presents three residential energy use datasets collected within the COFACTOR project [12] and the Research Centre on Zero Emission Neighbourhoods in Smart Cities (FME ZEN) [13]. Each dataset includes human-readable txt files with a consistent structure containing building information, hourly electricity and heating energy use, and corresponding weather data, spanning up to four years per building. The first dataset covers 29 apartment blocks with hourly electricity and delivered heat data. The second includes 20 files from Risvollan borettslag in Trondheim, comprising heating and electricity data for entire blocks and heating centrals, plus sub-metered electricity data from 1058 individual apartments. The third contains 194 files with smart meter data from single-family houses, cabins, and apartments equipped with Sikom home energy management systems.

## 3 Data Description

This manuscript is a data descriptor of a data product with three different datasets. Each of the datasets consist of several human readable comma-separated .txt files with residential building energy use data, including building information data, energy time series data and weather data from residential buildings in Norway. All the txt-files in the three different datasets follow the same structure. The time series data have a duration between 1–4 years per building with hourly resolution. The three datasets are summarized as follows:

**Dataset 1:** 29 files. Energy use data (heating and electricity) for condominiums of apartment blocks connected to 29 different heating centrals, with one file per heating central.
**Dataset 2:** 20 files. Energy use data (heating and electricity) for full apartment blocks and heating centrals as well as individual apartment electricity meters from 1058 apartments in Risvollan borettslag, a large condominium in Trondheim, Norway.
**Dataset 3:** 194 files. Smart meter data from 194 individual single-family houses, cabins and apartments collected through their Sikom home energy management systems.

Table 1 contains an overview of the datasets and the files in the datasets. The datasets and files are more thoroughly described later in this data descriptor.

**Table 1:** Characteristics of apartment buildings in the COFACTOR dataset.

	IDs	Description/ Location	N Files	Location	B. cat	Year of const-ruction	Heating	Floor area	N buildings	N units	Data period
Dataset 1	6470–6473	4 heating centrals with a total of 12 apartment blocks in Bærum	4	Bærum	Apt	1953/ 1954/1957	GSHP+EB+EFH or EB+EHW+EFH	20 321	12	252	2019–2022
	6474–6478	5 apartment blocks in the North-East of Oslo	5	Oslo	Apt	1974	ASHP+EHW+ EH+EFH	19 137	5	242	2019–2022
	6479–6495	17 apartment blocks in the east of Oslo (1 file per block)	17	Oslo	Apt	1966	DH	56 648	17	578	2019–2022
	6499	7 apartment blocks in the north of Oslo connected to 1 heating central	1	Oslo	Apt	1945	GSHP+EB+EHW	17 622	7	355	2019–2023
	6892–6893	2 apartment blocks in the north of Oslo (1 file per block)	2	Oslo	Apt	2018/2019	DH+EFH	13 523	2	155	2020–2023
Dataset 2	6449–6468	Large condominium with several heating stations and buildings	20	Trondheim	Apt	1972	DH+EFH	93 707	-	1058	2018–2022
Dataset 3	6698–6891	5 apartments (1 file each)	5	Norway	Apt	1985–2014	See own section.	507	-	5	Variable between 2021 and 2023
		154 cabins (1 file each)	154	Norway	Cab	1940–2018		20 218	154	154	
		35 houses (1 file each)	35	Norway	Hou	1940–2014		6 729	35	35	

*N*=number of. B.cat = Building category. Apt = Apartment or apartment block. Cab = Cabin. Hou = Single-family house.

ASHP = air source heat pump, DH = district heating, GSHP = ground source heat pump, EB = electric boiler, EFH = electric floor heating, EHW = electric hot water heater.

### 3.1 Contributions and clarifications

The datasets presented in this data descriptor provide novel energy use data from several residential buildings and units in Norway, including apartments, single family houses, and cabins. The energy use datasets are combined with complementary building and energy system information data. Comparable open datasets that combine hourly main meter data for electricity and heating with sub metered data, as well as energy use data from Norwegian cabins, are scarce. The structure of the datasets are similar to the structure of the datasets described in [14] and [15] and due to this, the sub-chapter on file-structure may have several similarities with these data descriptors. Although the overall file structure is similar across the three data descriptors, the datasets all contain primary energy use data that originate from different data owners, cover different building categories (public buildings, schools, and residential buildings), were collected at different times, and were supported by different research projects. The energy use data from the datasets constitute primary data collected directly from, or in collaboration with, the building owners for datasets 1 and 2, and from the home energy management provider Sikom for dataset 3, with explicit permission from the homeowners. Prior to the publication of the datasets described in the Data Descriptor, these data were private and not publicly available.

The level of raw data that can be made publicly available differs between the datasets due to data ownership agreements and GDPR considerations. For datasets 1 and 2, the raw data originally consisted of energy use measurements at the individual apartment level. However, in accordance with legal considerations in the energy companies, and in agreement with building owners, only data aggregated to the level of full apartment buildings could be released, in order to prevent re-identification of individual residential units. For dataset 3, raw energy use data were collected with explicit consent from homeowners for anonymized publication for research purposes. To protect their privacy, building information data have been anonymized, for example by resetting the location in from postal code to municipality. The published datasets therefore represent the highest possible level of raw data that can be shared while remaining compliant with GDPR and data owner agreements.

### 3.2 Repository

The data are stored in a repository organized as an open access Data Product in the data.sintef.no data platform (https://data.sintef.no/). A direct link to the repository is here: https://data.sintef.no/product/dp-b750d103-a434–45c2-ba5e-1af13f0a866f

The Data Product is called “COFACTOR” and contains a description of the datasets and a link to each of them, also called “Data features”. A summary of the different datasets/data features are given in Table 2. The files are human-readable, and the data product also contain a script with python code for reading the .txt files that may be used by others to efficiently read the files into data frames if using python. Due to GDPR considerations and data sharing agreements with the data providers, it is not possible to share all internal data processing code.

**Table 2:** Overview of the datasets and structure within the COFACTOR data product.

Reference	Data feature name	Files	Energy data available
Dataset 1	COFACTOR-Residential Dataset 1	Zip-folder with 29 .txt-files + README.txt.	Apartment blocks with some sub-meters in Oslo and Bærum.
Dataset 2	COFACTOR-Residential Dataset 2	Zip-folder with 20 .txt-files + README.txt.	Apartment blocks with main meters for heating and single meters per apartment in Trondheim
Dataset 3	COFACTOR-Residential Dataset 3	Zip-folder with 194 .txt-files + README.txt.	Main meters from 194 individual single-family houses, cabins and apartments.
Code	Python code for reading treASURE-files (building files with building information data and energy use data)	1 .py file for reading the .txt files with building information data and time series data to data frames.	-
Survey	Dataset 3: Anonymized survey and responses	1 .csv file with survey questions and answers.	-

The Data Features/datasets each have their own websites within the data product. Each of the datasets contain a dataset description as well as two files:

1) COFACTOR_Res_DatasetX_files.zip: A zip-folder for Dataset X containing several txt files, one per building or heating central. Each file contains building information data and hourly time series data. The txt files are named “uniqueID” to preserve anonymity and a unique ID.
2) README.txt: Description of dataset, txt-files and abbreviations in the txt files.

### 3.3 File structure

**Fig. 1:**
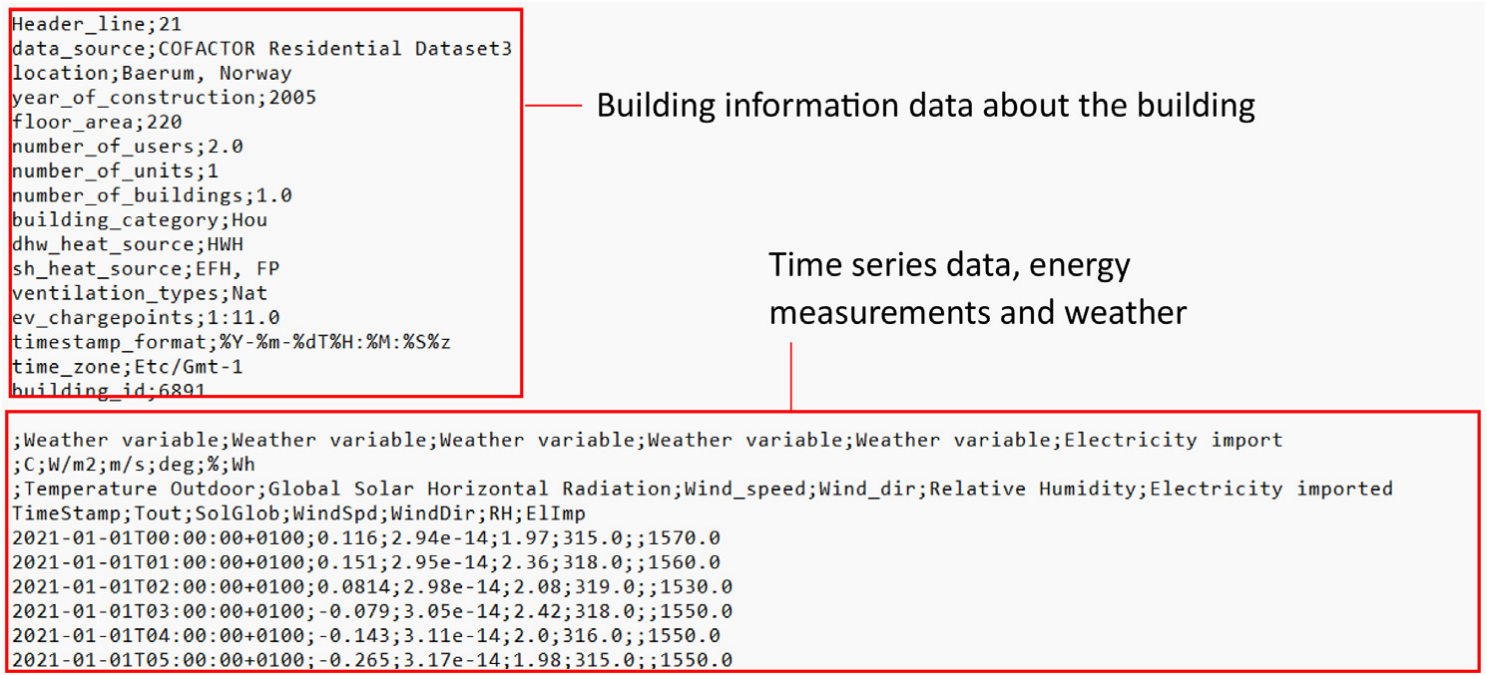
Example of the txt-file outline. The files in all of the datasets consists of one part with building information data and one part with time series data.

All files in the zip-folder within each of the datasets are txt-files and follow the same structure as shown in Fig. 1. Each of the txt files consist of two main parts; the first lines contain building information data, while the second part consist of the building time series data. The building data contains information about the buildings including the type of building, its size, and number of units for apartment blocks. The time series data contains 4 rows of headers, including the long name, unit, description and short name. The time series index contains timestamps formatted as %Y- %m- %dT %H: %M: %S%z and comply with ISO 8601. The timestamps are given in local normal time: CET (UTC+01:00). The time series is left-labelled, meaning that a timestamp such as 2018–01–01T00:00:00 + 01:00 represents the use during the hour 00:00–01:00. The actual local time zone of the location is “Europe/Oslo”.

Table 3 describes all the available building information data parameters, descriptions and abbreviations available in the txt-files.

**Table 3:** Characteristics of apartment buildings in the COFACTOR dataset.

Building information data parameter	Description	Data type	Example
Header_line	First line of measurements (for reading the txt)	Int	39
location	Location of the building	Str	Trondheim, Norway
year_of_construction	Year of construction	Int	1975
floor_area	Floor area in square meters	Int	130
number_of_units	Number of units in the building, e.g. the number of apartments in the building.	Int	1
number_of_users	Number of users (residents)	Int	2
number_of_buildings	Number of buildings on the lot	Int	1
building_category	Building category abbreviation. Apartment block or apartment: ‘Apt’ Single-family house: ‘Hou’ Cabin: ‘Cab’	Str	‘Hou’
notes	Any notes/additional information about the building and energy data	Str	‘Description’
dhw_heat_source	Type(s) of heating technology for hot water heating. See table below for available options.	Str	'HWH'
sh_heat_source	Type(s) of heating technology for space heating. See table below for available options.	Str	‘A2A, EH, EFH, FP’
pv	Photovoltaic system inverter capacity (kW)	Str or float	‘None’ or ‘10.4’
timestamp_format	Format for the timestamp in the time series	Str	‘ %Y- %m- %dT %H: %M: %S%z’
time_zone	Time zone	Str	‘Etc/Gmt-1′
building_id	Id of the building	Str	‘6730′
data_source	Description of the data source	Str	‘COFACTOR Residential Dataset3’
ev_chargepoints	Number of chargers and their capacity	Int:Float	1:3.6
ventilation_types	Ventilation types. Nat = Natural ventilation, Mech = Mechanic exhaust, CAV_fxd = Constant Air Volume fixed	Str	“Nat”

For heating of space heating (sh_heat_source) or domestic hot water (dhw_heat_source), the following options for heating technologies are available for the buildings, as given in Table 4. The buildings can use one or more heating appliances for each heating purpose.

**Table 4:** Abbreviations of heating types for the different energy services in the file building information data.

Option name	Option description	Available for energy services
A2A	Air to air heat pump	SH
ASHP	Air source heat pump	SH, DHW
DH	District heating	SH, DHW
EB	Electric boiler	SH, DHW
EFH	Electric floor heater	SH
EH	Electric heater	SH
FP	Fireplace	SH
GSHP	Ground source heat pump	SH, DHW
HWH	Hot water heater	DHW

SH = Space heating, DHW = domestic hot water heating.

Each .txt-file contain time-series data which has several columns, including energy main meters, energy sub-meters and weather data. Available column names, their descriptions and units are given in Table 5.

**Table 5:** Time series column names.

Column name	Description	Measurement category	Unit
TimeStamp	Time stamp in local UTC time	Time	Time
Tout	Temperature Outdoor	Weather variable	C
SolGlob	Global Solar Horizontal Radiation	Weather variable	W/m^2^
WindSpd	Wind speed	Weather variable	m/s
WindDir	Wind direction	Weather variable	deg
RH	Relative humidity	Weather variable	%
ElAux	Auxiliary electricity use	Electricity use	Wh
ElBoil	Electricity for electric boilers	Electricity use	Wh
ElEV	Electricity for EV-chargers	Electricity use	Wh
ElHP	Electricity for heat pumps	Electricity use	Wh
ElHWH	Electricity for electric hot water heaters	Electricity use	Wh
ElHt	Electricity for electric heaters	Electricity use	Wh
ElImp	Electricity imported to the building.	Electricity Import (main meter)	Wh
ElImp_apt	Electricity imported, sum of AMS meters of apartments.	Electricity Import	Wh
ElMix	Electricity for mixed purposes (heating and other)	Electricity use	Wh
ElMix_exp	Electricity exported from a meter with mixed purposes	Electricity export	Wh
ElOth	Electricity for other purposes	Electricity use	Wh
ElPlug	Electricity plug loads	Electricity use	Wh
HtDH	Heating from district heating	Heat use (main meter)	Wh
HtDHW	Heating energy for Domestic Hot Water	Heat use	Wh
HtHP	Heat from heat pumps	Heat production	Wh
HtSpace	Heating energy for space heating.	Heat use	Wh
HtTot	Total heating energy. HtTot = HtSpace + HtDHW	Heat use (sometimes main meter)	Wh

## 4 Experimental Design, Materialsl and Methods

All of the three datasets and files contain building information data as well as time series data from weather and energy use. This section describes the scope of the data collection, the data collection process for different datasets and some insights into the data within each dataset. The collection process for each dataset is described further in the sections below. For most of the buildings, the type, calibration, and accuracy of sub-meters vary and are not documented. Main electricity meters and district heating meters’ calibration and accuracy are regulated by law. Generally, the processing of the data from the different data sources includes the selection of suitable buildings and meters to fit the study scope, relabelling of meters, unit conversions, combining weather data with energy use data, resetting timestamps to UTC, converting files to standard file formats, as well as simple data cleaning as described in the following section. These steps were performed using R (v3.x) and Python (v3.11). The energy use data are recorded at native equidistant hourly intervals by the underlying metering infrastructure, in accordance with Norwegian main meter requirements and have not been resampled or otherwise temporally processed.

### 4.1 Scope of data collection

The datasets were collected through the COFACTOR project and FME ZEN, to provide new knowledge on the energy use patterns of Norwegian residential buildings. In COFACTOR, the data are used to provide new and updated coincidence factors and standard load profiles for different building categories, as well as to suggest a methodology for estimating the peak load of buildings. In FME ZEN, the data was analysed to understand the energy profiles and electricity flexibility potential in Norwegian apartment buildings with electric vehicle charging.

### 4.2 Weather data

Each building file includes time series data for both weather and energy use. Meteorological weather data has been obtained for the geographical locations of the buildings from the MET Nordic dataset provided by the Norwegian Meteorological Institute [16]. The building locations are identified using zip codes, and the geographical coordinates are then sourced from a database created by Erik Bolstad (https://www.erikbolstad.no/postnummer-koordinatar/).

Table 6 Weather data parameters and corresponding name in source database.

**Table 6:** presents the collected weather data, listing both the dataset names and their corresponding names in the MET Nordic dataset.

Name	Explanation	Unit	MET Nordic name	Comment
Tout	Temperature Outdoor	C	air_temperature_2m	-
SolGlob	Global Solar Horizontal Radiation	W/m^2^	integral_of_surface_downwelling_ shortwave_flux_in_air_wrt_time	Shifted backwards by one hour to align with “left label”.
WindSpd	Wind speed	m/s	wind_speed_10m	-
WindDir	Wind direction	deg	wind_direction_10m	-
RH	Relative humidity	%	relative_humidity_2m	

### 4.3 DATASET 1: hourly electricity and heating data from apartment buildings

Dataset 1 consist of hourly electricity use and heating energy use data from apartment blocks in 29 locations in Oslo and Bærum with one file per heating central/location. The data in Dataset 1 was collected from 8 different housing associations which includes in total 43 apartment blocks and 2330 apartments. More details about the different files and apartment blocks are given in Table 7. The data was collected in co-operation with Dråpe Entreprenør AS, the local DSO Elvia AS, the local district heating provider Hafslund Celsio AS and the building owners/housing associations

**Table 7:** Overview of the files in Dataset 1 including Building IDs with most relevant building information data.

ID	Loc	Year of const-ruction	Floor area	N units	N build-ings	SH Heat Source	DHW Heat Source	Energy columns
6470	Bærum	1954	11,011	143	12	GSHP, EB, EFH	GSHP, HWH	ElOth’, ‘ElHt’, ‘ElImp’, ‘ElImp_apt’
6471		1957	3096	36	3	EB, EFH	HWH	ElMix’, ‘ElOth’, ‘ElHt’, ‘ElImp’, ‘ElImp_apt'
6472		1957	2064	24	2	EB, EFH	HWH	ElHt’, ‘ElPlug’, ‘ElOth’, ‘ElEV’, ‘ElImp’, ‘ElImp_apt'
6473		1953	4150	49	3	GSHP, EB, EFH	GSHP, HWH	ElOth’, ‘ElEV’, ‘ElHt’, ‘ElImp’, ‘ElImp_apt'
6474	NE Oslo	1974	3163	40	1	EH, EFH	ASHP, HWH	ElMix’, ‘ElImp’, ‘ElImp_apt'
6475		1974	4191	53	1	EH, EFH	ASHP, HWH	ElMix’, ‘ElImp’, ‘ElImp_apt'
6476		1974	5457	69	2	EH, EFH	ASHP, HWH	ElMix’, ‘ElImp’, ‘ElImp_apt'
6477		1974	4033	51	1	EH, EFH	ASHP, HWH	ElMix’, ‘ElImp’, ‘ElImp_apt'
6478		1974	2293	29	1	EH, EFH	ASHP, HWH	ElMix_exp’, ‘ElMix’, ‘ElEV’, ‘ElImp’, ‘ElImp_apt'
6479	E Oslo	1966	2304	24	1	DH	DH	ElOth’, ‘ElImp’, ‘HtDH’, ‘HtSpace’, ‘HtDHW’, ‘ElImp_apt'
6480			2880	30	1	DH	DH	ElOth’, ‘ElImp’, ‘HtDH’, ‘HtSpace’, ‘HtDHW’, ‘ElImp_apt'
6481			2304	24	1	DH	DH	ElOth’, ‘ElImp’, ‘HtDH’, ‘HtSpace’, ‘HtDHW’, ‘ElImp_apt'
6482			2880	30	1	DH	DH	ElOth’, ‘ElEV’, ‘ElImp’, ‘HtDH’, ‘HtSpace’, ‘HtDHW’, ‘ElImp_apt'
6483			2880	30	1	DH	DH	ElOth’, ‘ElImp’, ‘HtDH’, ‘HtSpace’, ‘HtDHW’, ‘ElImp_apt'
6484			2880	30	1	DH	DH	ElOth’, ‘ElImp’, ‘HtDH’, ‘HtSpace’, ‘HtDHW’, ‘ElImp_apt'
6485			2880	30	1	DH	DH	ElOth’, ‘ElImp’, ‘HtDH’, ‘HtSpace’, ‘HtDHW’, ‘ElImp_apt'
6486			2880	30	1	DH	DH	ElOth’, ‘ElImp’, ‘HtDH’, ‘HtSpace’, ‘HtDHW’, ‘ElImp_apt'
6487			2880	30	1	DH	DH	ElEV’, ‘ElOth’, ‘ElImp’, ‘HtDH’, ‘HtSpace’, ‘HtDHW’, ‘ElImp_apt'
6488			2880	30	1	DH	DH	ElOth’, ‘ElImp’, ‘HtDH’, ‘HtSpace’, ‘HtDHW’, ‘ElImp_apt'
6489			5000	50	1	DH	DH	ElOth’, ‘ElImp’, ‘HtDH’, ‘HtSpace’, ‘HtDHW’, ‘ElImp_apt'
6490			3000	30	1	DH	DH	ElOth’, ‘ElImp’, ‘HtDH’, ‘HtSpace’, ‘HtDHW’, ‘ElImp_apt'
6491			5000	50	1	DH	DH	ElOth’, ‘ElImp’, ‘HtDH’, ‘HtSpace’, ‘HtDHW’, ‘ElImp_apt'
6492			3000	30	1	DH	DH	ElAux’, ‘ElOth’, ‘ElImp’, ‘HtDH’, ‘HtSpace’, ‘HtDHW’, ‘ElImp_apt'
6493			5000	50	1	DH	DH	ElOth’, ‘ElImp’, ‘HtDH’, ‘HtSpace’, ‘HtDHW’, ‘ElImp_apt'
6494			3000	30	1	DH	DH	ElOth’, ‘ElImp’, ‘HtDH’, ‘HtSpace’, ‘HtDHW’, ‘ElImp_apt'
6495			5000	50	1	DH	DH	ElOth’, ‘ElImp’, ‘HtDH’, ‘HtSpace’, ‘HtDHW’, ‘ElImp_apt'
6499	N Oslo	1945	17,622	355	7	GSHP, EB	GSHP, EB, HWH	ElOth’, ‘ElMix’, ‘ElImp’, ‘HtDHW’, ‘HtSpace’, ‘ElBoil’, ‘HtTot’, ‘ElHWH’, ‘ElHP’, ‘HtHP’, ‘ElImp_apt'
6892	Oslo	2019	6926	77	1	DH, EFH	DH	HtDH’, ‘ElImp’, ‘ElImp_apt'
6893		2018	6597	78	1	DH, EFH	DH	ElOth’, ‘ElAux’, ‘ElImp’, ‘ElImp_apt'

All the locations (except ID 6474–6478) have centralized space heating and domestic hot water (DHW) production, making it possible to fully distinguish between heating and electric specific energy use in these apartment blocks. The exceptions from this are the apartments with electric floor heating (EFH) in their bathrooms, which is common in Norway even in apartment blocks with a central heating system. Here, the electricity for EFH is included in the apartment electricity use. The apartment blocks with IDs 6474–6478 have centralized DHW production, but the space heating demand is covered by electricity behind the apartment meters, making it impossible to exactly distinguish the electricity use for space heating from other electricity use in these apartments with the current set up.

Hourly electricity use measurements and fuse sizes were collected for all the apartment blocks, including separate electricity use meters for common areas and individual apartments, from the local DSO. The meters are from Aidon, and the data has hourly resolution. For privacy reasons, individual apartment electricity measurements were aggregated to total electricity for all the apartments served by the same heating centrals. This sum is labelled “ElImp_apt” in the files. The files also contain electricity use meters for the common areas in the apartment blocks which were labelled based on communication with the apartment block owners and manual interpretation of the data, including fuse size and energy use pattern.

For the apartment blocks with centralized electricity-based heating systems, the heating centrals are equipped with separate meters from the DSO that measure the electricity use for heating (“ElHt”). Users of the dataset should be beware that the seasonal coefficient of performance (SCOP) or efficiency of these electric heating centrals are unknown.

The file with ID 6499 has been treated differently than the other files as this apartment condominium had several additional heating energy meters installed by the building owner. These meters are less accurate than the meters installed by the DSO and DH companies. Also, the system of building 6499 is quite complex, with 8 ground source heat pumps with hot gas heat exchangers, an electric boiler and an oil boiler. Due to the placement of these heating energy meters, the “HtSpace” and “HtDHW” is calculated as combinations of multiple meters, which may be a source of errors. These measurements should therefore be used with care. If any of the datapoints in the combined meters were missing, the corresponding datapoints in the combined meters were also set as missing (NaN), This avoids the possibility of misleading values in combined meters. Except for the aggregation and combination of the heating sub-meters in 6499, the data from the sub- and main meters of Dataset 1 have not been cleaned.

For the buildings with district heating, district heating measurement data were collected from the local district heating company. In addition, for buildings 6479–6495 separate meters for DHW energy use (“HtDHW”) are installed. For these buildings sthe space heating energy use (“HtSpace”) is estimated by subtracting “HtDHW” from the total imported heating energy use from district heating (“HtDH”).

The measurement resolution of all energy use data collected from the electricity smart meters available in Dataset 1 are 1 Wh, while the data from the heating energy meters and electric sub-meters are 1 kWh.

**Fig. 2:**
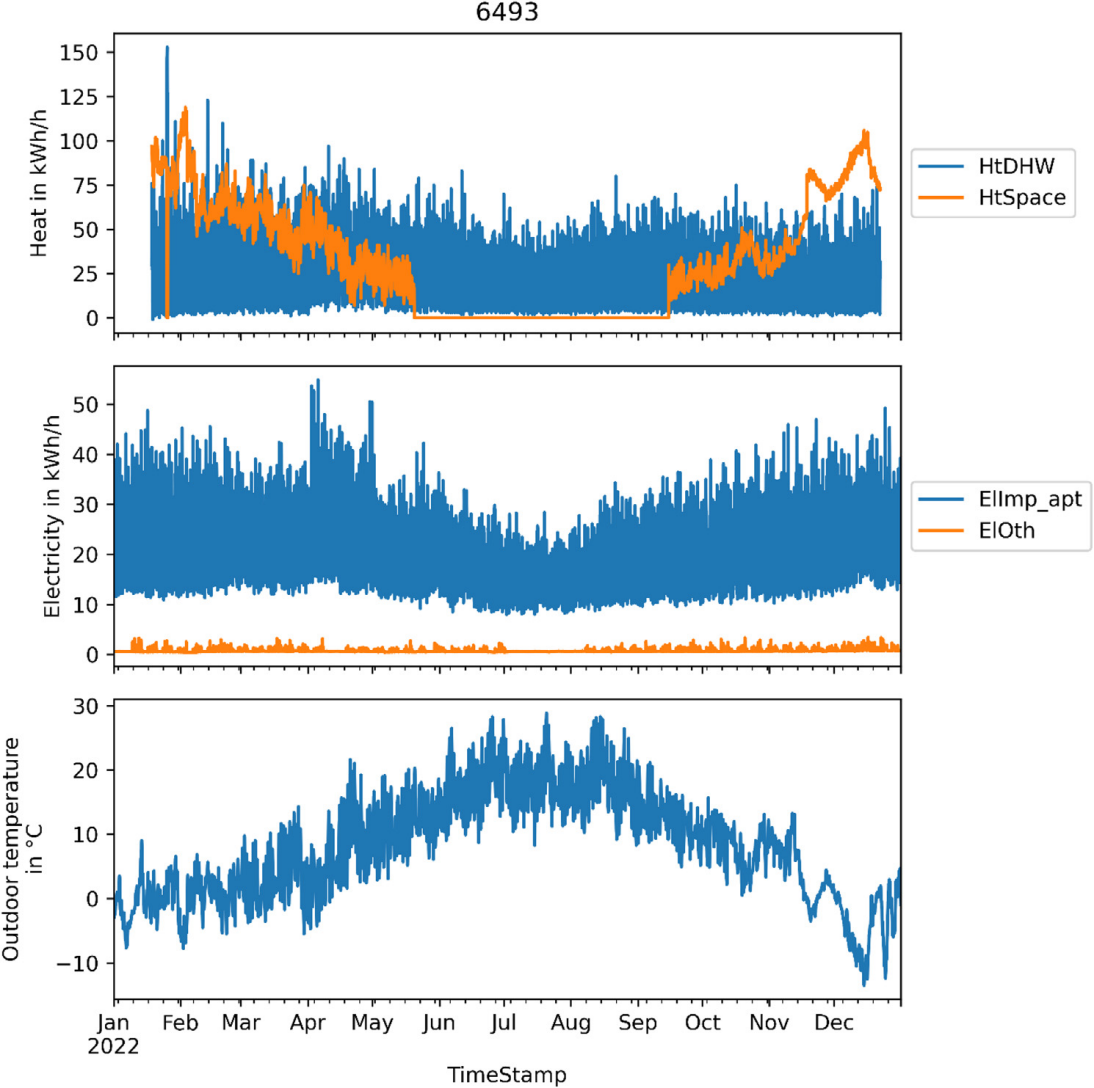
Example plot of heating and electricity energy use data for the apartment block with ID 6493.

Fig. 2 shows energy use data from one selected apartment block (6493). The top plot shows heating energy use, split in space heating energy use (“HtSpace”) and energy use for heating of domestic hot water (“HtDHW”). The space heating system is turned off in the summer. The middle plot shows the electricity use of the same building for the same time period, for the apartments (“ElApt”) and for the common areas (“ElOth”). The outdoor temperature is shown in the bottom plot.

### 4.4 DATASET 2: hourly electricity data from 1058 individual apartments and hourly heating data from 20 heating districts

Dataset 2 contains energy use data collected from Risvollan Borettslag which is a large housing association in Trondheim with 1058 apartments in 121 low-rise buildings. The buildings were constructed in 1972 and consist of apartments with 1 to 4 bedrooms and 53 to 107 m^2^ floor area (average 88.6 m^2^). In average, there are 1.8 residents per household. The buildings are connected to the local district heating network. District heating is used for both space heating and domestic hot water heating. The dataset is used in several research articles further describing the heating system [2], electricity use [3], simulation of photovoltaic (PV) electricity generation [4], building renovation [5], domestic hot water (DHW) use [6], Electric Vehicle (EV) charging [7,8,9], and energy flexibility potential [10,11], but has previously not been published.

Dataset 2 consist of 20 txt-files. Each of the files represent the apartment blocks connected to each of the 20 heating substations in the housing association. Each of the files contain building information data about the buildings connected to each central as well as energy use data which consist of total hourly electricity use for the apartments combined (“ElImp_Apt”), the total electricity use for the apartment blocks (“ElImp”), as well as individual meters for the electricity use in each apartment (“ElImp_X” where X refer to an anonymized apartment ID). 13 of the 20 files contain hourly measurements of energy use from district heating (“HtDH”) . In addition, the energy use data in some of the file contain sub-metered energy use for electric vehicle charging (“ElEV”), energy use for heating of domestic hot water heating (“HtDHW”) and electricity use for common areas (“ElOth”).

The following tables includes information about:

• Table 8: Building (file) IDs, number of buildings, floor area for the apartments, number of apartments, the start and end date of available data series, as well as the resolution steps for the district heating (HtDH) meter.

**Table 8:** Overview of the files in Dataset 2 including Building/file IDs, number of buildings, floor area for the apartments, number of apartments, the start and end date of available data series, as well as the resolution steps for the HtDH meter. (Dataset 2).

Building IDs	Buildings (n)	Floor area [m^2^]	Units (n)	Data series (start date, end date)						Resol. steps heating
				ElImp_apt	ElOth	ElEV	ElImp	HtDH	HtDHW	kWh
6449	5	4408	49	14.02.2018 31.12.2022	02.01.2018 31.12.2022	31.12.2017 31.12.2022	14.02.2018 31.12.2022	NA	NA	NA
6450	4	3114	34	31.12.2019 31.12.2022	31.12.2017 31.12.2022	NA	31.12.2019 31.12.2022	NA	NA	NA
6451	6	5258	56	31.12.2020 31.12.2022	08.02.2018 31.12.2022	08.02.2018 31.12.2022	31.12.2020 31.12.2022	31.12.2017 02.01.2023	NA	10
6452	9	5395	67	31.12.2019 31.12.2022	08.02.2018 31.12.2022	12.02.2018 31.12.2022	31.12.2019 31.12.2022	NA	NA	NA
6453	8	4938	55	31.12.2018 31.12.2022	08.02.2018 31.12.2022	08.02.2018 31.12.2022	31.12.2018 31.12.2022	NA	NA	NA
6454	10	6726	71	31.12.2020 31.12.2022	16.10.2018 31.12.2022	16.10.2018 31.12.2022	31.12.2020 31.12.2022	NA	NA	NA
6455	8	5783	65	31.12.2020 31.12.2022	31.12.2017 31.12.2022	NA	31.12.2020 31.12.2022	NA	NA	NA
6456	5	4758	56	31.12.2019 31.12.2022	19.04.2018 31.12.2022	NA	31.12.2019 31.12.2022	31.12.2017 02.01.2023	NA	10
6457	7	4758	50	31.12.2021 31.12.2022	31.12.2017 31.12.2022	31.12.2017 31.12.2022	31.12.2021 31.12.2022	29.06.2020 02.01.2023	NA	10
6458	5	3790	44	31.12.2019 31.12.2022	31.12.2017 31.12.2022	NA	31.12.2019 31.12.2022	31.12.2017 02.01.2023	NA	10
6459	6	3081	33	11.12.2018 31.12.2022	31.12.2017 31.12.2022	31.12.2017 31.12.2022	11.12.2018 31.12.2022	29.06.2020 02.01.2023	NA	10
6460	8	6933	73	31.12.2019 31.12.2022	31.12.2017 31.12.2022	31.12.2017 31.12.2022	31.12.2019 31.12.2022	NA	NA	NA
6461	5	4662	54	31.12.2020 31.12.2022	31.12.2017 31.12.2022	NA	31.12.2020 31.12.2022	31.12.2017 02.01.2023	NA	10
6462	5	4197	45	27.02.2018 31.12.2022	31.12.2017 31.12.2022	31.12.2017 31.12.2022	27.02.2018 31.12.2022	31.12.2017 02.01.2023	NA	10
6463	6	3680	43	31.12.2018 31.12.2022	31.12.2017 31.12.2022	NA	31.12.2018 31.12.2022	31.12.2017 02.01.2023	NA	10
6464	6	4887	62	13.12.2018 31.12.2022	31.12.2017 31.12.2022	NA	13.12.2018 31.12.2022	31.12.2017 02.01.2023	NA	10
6465	5	4507	54	31.12.2020 31.12.2022	25.01.2018 31.12.2022	NA	31.12.2020 31.12.2022	31.12.2017 02.01.2023	NA	10
6466	4	4149	48	04.11.2018 31.12.2022	23.01.2018 31.12.2022	NA	04.11.2018 31.12.2022	31.12.2017 02.01.2023	NA	10
6467	5	6139	74	31.12.2019 31.12.2022	23.10.2018 31.12.2022	30.11.2018 31.12.2022	31.12.2019 31.12.2022	31.12.2017 02.01.2023	20.09.2018 02.01.2023	10
6468	4	2544	25	31.10.2018 31.12.2022	26.02.2018 31.12.2022	26.02.2018 31.12.2022	31.10.2018 31.12.2022	29.06.2020 02.01.2023	NA	10
TOTAL	121	93,707	1058	20 IDs	20 IDs	11 IDs	20 IDs	13 IDs	1 ID
• Table 9: Building information data per file/Building ID, including number of users, floor area, number of apartment units and buildings.

**Table 9:** Building information data per Building ID, including number of users, floor area, number of apartment units and buildings. (Dataset 2).

Building ID	Floor area [m^2^]	N units	N buildings	N users
6449	4408	49	5	107
6450	3114	34	4	73
6451	5258	56	6	137
6452	5395	67	9	127
6453	4938	55	8	118
6454	6726	71	10	182
6455	5783	65	8	141
6456	4758	56	5	129
6457	4758	50	7	106
6458	3790	44	5	99
6459	3081	33	6	80
6460	6933	73	8	166
6461	4662	54	5	124
6462	4197	45	5	107
6463	3680	43	6	98
6464	4887	62	6	115
6465	4507	54	5	117
6466	4149	48	4	100
6467	6139	74	5	139
6468	2544	25	4	56
• Table 10: Distribution of different type of apartments, floor area, and number of bedrooms per file/Building ID.

**Table 10:** Distribution of different type of apartments, floor area, and number of bedrooms per Building ID. (Dataset 2).

Building ID groups	One-bedroom apt (52.9 m^2^ floor area)	Two-bedroom apt (83.5 m^2^ floor area)	Three-bedroom apt (104.8 m^2^ floor area)	Four-bedroom apt (107.2 m^2^ floor area)	Total apt (n)	Bedrooms (n)
6449, 6450	20	7	51	5	83	207
6451, 6452, 6453	52	16	102	8	178	422
6454	14		52	5	71	190
6455, 6456, 6457	40	26	101	4	171	411
6458	16		24	4	44	104
6459, 6460, 6461	32	22	90	16	160	410
6462, 6463, 6464	44	32	70	4	150	334
6465, 6466, 6467, 6468	44	70	66	21	201	466
	262	173	556	67	1058
• Table 11: Floor area for garages per file/Building ID.

**Table 11:** Floor area for garages per Building ID. (Dataset 2).

Building ID	Garages in basements (n) (floor area (m^2^))
6449	2 (1903)
6452	2 (1087)
6453	1 (906)
6451	2 (1359)
6454	2 (1632)
6456	5 (3442)
6460	4 (2716)
6459	1 (362)
6462	3 (2809)
6467	1 (1087)
6468	1 (453)
	24 (16,397)
• Table 12: Number of residents and age group in the heating districts, from [17].

**Table 12:** Number of residents and age group in the heating districts. (Dataset 2).

Building IDs	Age group (number of residents (female, male))									Residents (n)
	< 10	10 - 19	20 - 29	30 - 39	40 - 49	50 - 59	60 - 69	70 - 79	> 80	
6449	13 (4, 9)	10 (6, 4)	18 (7, 11)	17 (9, 8)	13 (7, 6)	11 (6, 5)	9 (5, 4)	14 (8, 6)	2 (1, 1)	107 (53, 54)
6450	9 (4, 5)	10 (5, 5)	4 (4, 0)	16 (8, 8)	10 (3, 7)	7 (3, 4)	7 (3, 4)	6 (3, 3)	4 (3, 1)	73 (36, 37)
6451	26 (14, 12)	11 (7, 4)	14 (10, 4)	19 (9, 10)	27 (14, 13)	8 (4, 4)	15 (10, 5)	14 (7, 7)	3 (2, 1)	137 (77, 60)
6452	9 (8, 1)	14 (6, 8)	14 (10, 4)	14 (6, 8)	17 (9, 8)	21 (6, 15)	11 (6, 5)	18 (10, 8)	9 (6, 3)	127 (67, 60)
6453	14 (8, 6)	12 (4, 8)	11 (4, 7)	15 (8, 7)	15 (7, 8)	13 (6, 7)	11 (6, 5)	17 (10, 7)	10 (6, 4)	118 (59, 59)
6454	25 (14, 11)	26 (10, 16)	17 (9, 8)	29 (15, 14)	23 (10, 13)	27 (15, 12)	14 (9, 5)	9 (7, 2)	12 (5, 7)	182 (94, 88)
6455	15 (10, 5)	24 (14, 10)	12 (7, 5)	24 (14, 10)	26 (14, 12)	22 (13, 9)	10 (6, 4)	7 (4, 3)	1 (1, 0)	141 ()
6456	14 (7, 7)	14 (4, 10)	9 (6, 3)	9 (5, 4)	14 (7, 7)	20 (14, 6)	13 (6, 7)	13 (7, 6)	0 (0, 0)	106 (56, 50)
6457	20 (11, 9)	11 (4, 7)	25 (14, 11)	24 (11, 13)	16 (10, 6)	14 (7, 7)	8 (5, 3)	10 (6, 4)	1 (1, 0)	129 (69, 60)
6458	16 (10, 6)	10 (4, 6)	8 (2, 6)	12 (8, 4)	8 (3, 5)	13 (7, 6)	12 (9, 3)	13 (6, 7)	7 (4, 3)	99 (53, 46)
6459	18 (7, 11)	3 (2, 1)	12 (9, 3)	15 (8, 7)	5 (2, 3)	4 (2, 2)	9 (5, 4)	10 (5, 5)	4 (2, 2)	80 (42, 38)
6460	18 (7, 11)	14 (9, 5)	24 (14, 10)	22 (10, 12)	27 (15, 12)	18 (8, 10)	17 (11, 6)	20 (11, 9)	6 (2, 4)	166 (87, 79)
6461	18 (8, 10)	17 (7, 10)	18 (9, 9)	24 (14, 10)	21 (11, 10)	10 (5, 5)	8 (5, 3)	6 (4, 2)	2 (2, 0)	124 (65, 59)
6462	18 (8, 10)	10 (5, 5)	14 (4, 10)	18 (12, 6)	12 (6, 6)	14 (9, 5)	9 (6, 3)	8 (4, 4)	4 (3, 1)	107 (57, 50)
6463	11 (5, 6)	13 (5, 8)	12 (5, 7)	18 (9, 9)	6 (5, 1)	16 (8, 8)	14 (9, 5)	5 (4, 1)	3 (2, 1)	98 (52, 46)
6464	14 (7, 7)	2 (1, 1)	22 (9, 13)	15 (7, 8)	10 (3, 7)	19 (15, 4)	16 (12, 4)	9 (5, 4)	8 (5, 3)	115 (64, 51)
6465	17 (6, 11)	13 (2, 11)	11 (6, 5)	17 (7, 10)	17 (10, 7)	19 (7, 12)	14 (8, 6)	8 (4, 4)	1 (1, 0)	117 (51, 66)
6466	10 (4, 6)	5 (3, 2)	9 (4, 5)	16 (7, 9)	14 (8, 6)	21 (12, 9)	13 (6, 7)	12 (7, 5)	0 (0, 0)	100 (51, 49)
6467	15 (9, 6)	12 (10, 2)	19 (9, 10)	22 (11, 11)	18 (10, 8)	11 (7, 4)	19 (11, 8)	17 (11, 6)	6 (3, 3)	139 (81, 58)
6468	8 (3, 5)	8 (4, 4)	1 (1, 0)	8 (4, 4)	4 (2, 2)	11 (6, 5)	4 (3, 1)	8 (3, 5)	4 (2, 2)	56 (28, 28)
Total	308 (154, 154)	239 (112, 127)	274 (143, 131)	354 (182, 172)	303 (156, 147)	299 (160, 139)	233 (141, 92)	224 (126, 98)	87 (51, 36)	2321 (1225, 1096)

Building information data for each file (provided in the txt-files and described in Table 8, Table 9, Table 10, and Table 11) was collected from the housing cooperative Risvollan and their energy management system contractors. The electricity use data from the individual apartments are anonymized due to privacy reasons, and the exact address and size of each apartment is therefore unknown. Energy use data in the txt-files was collected from the local DSO (electricity use data from smart meters) and the energy management system of the housing cooperative (heating energy use data).

The quality of electricity use data from smart meters is regulated by law and is generally of high quality. All energy use measurements from smart meters are raw data. Some of the electricity smart meters still had missing data periods, and those hours were set to NaN in the files.

The measurement resolution of the electricity use meters including ElImp, ElImp_X, ElImp_apt, ElEV and ElOth is 1 Wh. The measurement resolution of the heating meters (HtDH and HtDHW) differed between the buildings is 10 kWh.

For the 20 substations, the meters for district heating energy use had a resolution of 10 kWh per hour for 13 of the buildings, whereas the remaining 7 substations recorded this energy use data at a coarser resolution of 100 kWh per hour. A measurement resolution of “10″ (kWh), means that the meter only registers energy use events when the accumulated energy use surpasses multiples of 10 kWh. Consequently, the hourly energy use between each 10 kWh of energy use becomes 0, causing “jumps” in the energy use. Based on the evaluation of measurement resolution and data quality, all the 7 substations with 100 kWh resolution were excluded from the final dataset.

### 4.5 DATASET 3: hourly electricity data and survey responses from about 35 houses and 150 cabins with smart electricity management

Dataset 3 contains 194 files with building information data and smart meter data from 194 individual single-family houses, cabins and apartments collected in co-operation with Sikom AS and from their home energy management system. Sikom Connect AS is a Norwegian company specializing in smart control solutions for heating, electricity, and security in homes and cabins.

Sikom customers were invited to share their hourly, total electricity use data, and to participate in a survey about their residential units for research purposes - voluntarily and anonymously. The questionnaire was developed by researchers at SINTEF and distributed digitally by Sikom to its customers. The survey was sent to all Sikom customers equipped with Sikom energy use meters. No preselection of participants was performed. The survey invited the customers to share their data and collected information on building characteristics, including building type, floor area, construction period, and implemented energy efficiency measures, as well as household size. In addition, respondents reported on their heating systems for space heating and domestic hot water, ventilation type, use of solid fuel heating, and the presence and characteristics of electric vehicle charging and rooftop photovoltaic systems. The questions were primarily closed-ended with predefined answer categories, supplemented by optional free text fields where additional detail was needed. The questions given in the survey are given in Table 13. The questions were designed to be direct and unambiguous, focusing on factual characteristics rather than subjective assessments in order to minimize response bias. Due to GDPR considerations, Sikom was responsible for distributing the questionnaire, collecting responses, linking survey answers to the corresponding energy use data, and anonymizing the dataset prior to transfer to the authors of this data descriptor. Relevant information from the survey responses were subsequently extracted and used to populate the building information in each building file in dataset 3. A summary of the building information data statistics from the 194 files are given Table 9. The original questionnaire was developed in Norwegian and is published in the data repository described in the Data Description section.

**Table 13:** Translated questions from the Sikom user survey and types of answers given by the respondents.

	Type of answer
2. What is the postal code of the property?	Numerical
3. What is the property type?	5 options
4. What is the area of the property?	Numerical
5. How many people live in the property?	1–9+
6. What is the construction year of the property?	10 options
7. Have energy efficiency measures been implemented?	9 options (multiple)
8. How is the property heated?	
8c. Is the fireplace used for heating?	4 options
9. What heating sources are used for heating domestic hot water?	5 options
10. How is your property ventilated?	4 options
11. How do you charge your car at your property?	3 options
11bWhat is the maximum power your electric car charges with at your property? (choose the closest option)	5 options
11c. Do you control the charging of your electric car? (e.g., plug in the afternoon but charging starts at night)	3 options
11d If you have an electric car: What kind of car do you have? (model and year of the car(s) used most frequently)	Free text
12. Are solar panels installed and connected to your electricity meter?	Yes/no
21b If yes: What is the installed capacity in kW?	Numerical
13. Do you use Sikom's options for controlling energy use?	3 options
14. Is there any other information you think is relevant to the energy use in your property?	Free text

239 Sikom customers volunteered to share their electricity use data and filled in the survey. However, 45 buildings were excluded due to limited data available (see considerations below), yielding a total of 194 building files (5 apartments, 154 cabins and 35 houses) in dataset 3.

The duration of the time series data within each files varies between a few months to two years. Each of the 194 files in Dataset 3 contain data from one electricity meter “ElImp”, which shows the hourly imported electricity use to the building. The measurement resolution of these electricity use meters is 10 Wh.

As the Sikom electricity meters are not used for billing purposes, and thereby not under the same quality regulations as DSO meters, the electricity data has been slightly cleaned. The following cleaning process has been applied:

For timestamps where more than 50 % of all meters/files has a value of exactly 0, it was assumed that something was wrong with the energy collection system, and all metering points were set to NaN for these time steps.
The core purpose of the project where the data was collected, was to evaluate typical energy use patterns and peak loads of residential buildings. It was considered that a minimum amount of data was required to evaluate this. 2000 hours corresponds to approximately 3 months. Buildings with less than 2000 valid measurement points were hence excluded.

**Fig. 3:**
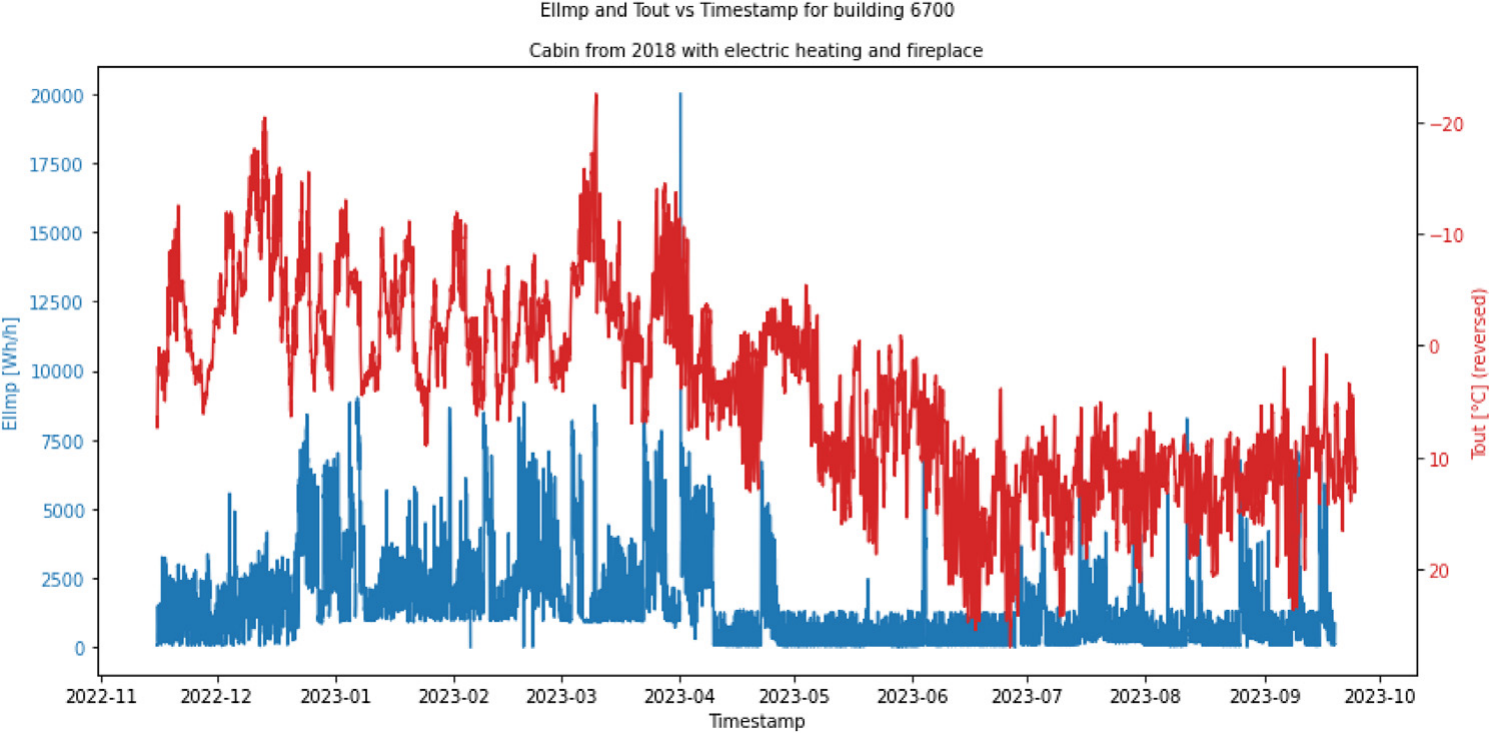
Tout and ElImp plotted against the TimeStamp for building 6700 which is a cabin from 2018 with electric heating and a fireplace.

Fig. 3 shows an example of data plotted for one of the buildings, ID 6700. This is a cabin from 2018 with electric heating and a fireplace at 146 m^2^ and with an EV-charger. The building has about 10 months of energy use data. The figure shows that there is variable electricity use during the year, with variation in energy use both dependent on the season/outdoor temperature as well as intermittent occupancy.

The following figures includes the following information about the buildings in Dataset 3:

• Table 14*: Average, maximum and minimum floor area* in the residential units, split by building type.

**Table 14:** Average, maximum and minimum floor area given in the building information data for the Sikom buildings in Dataset 3 per building category.

	Apt	Cab	Hou
Average [m^2^]	101	131	192
Max [m^2^]	200	902	350
Min [m^2^]	50	50	65
• Figure 4: Heating appliance *combinations* present in the residential units, split by building type.

**Fig. 4:**
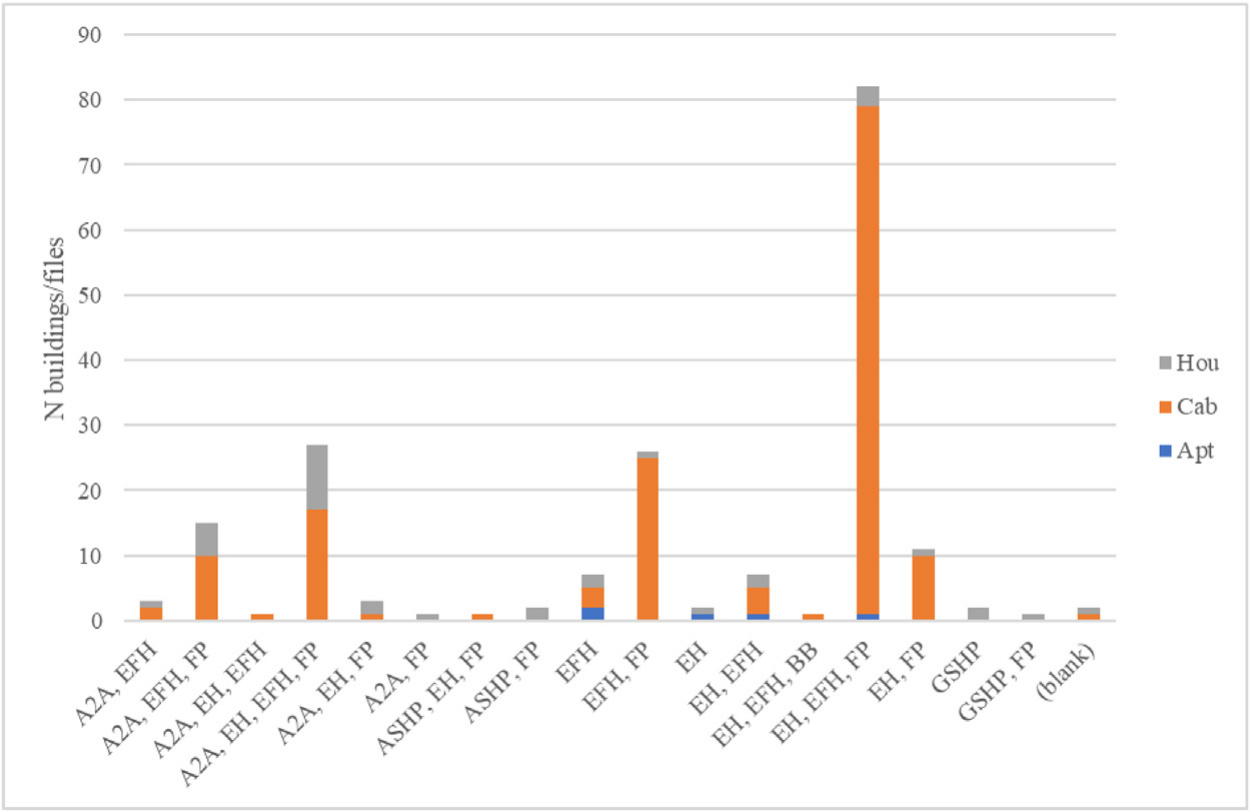
Space heating appliance combinations given in the building information data for the Sikom buildings in Dataset 3 per building category.
• Figure 5: Space heating appliances present in the residential units, split by building type.

**Fig. 5:**
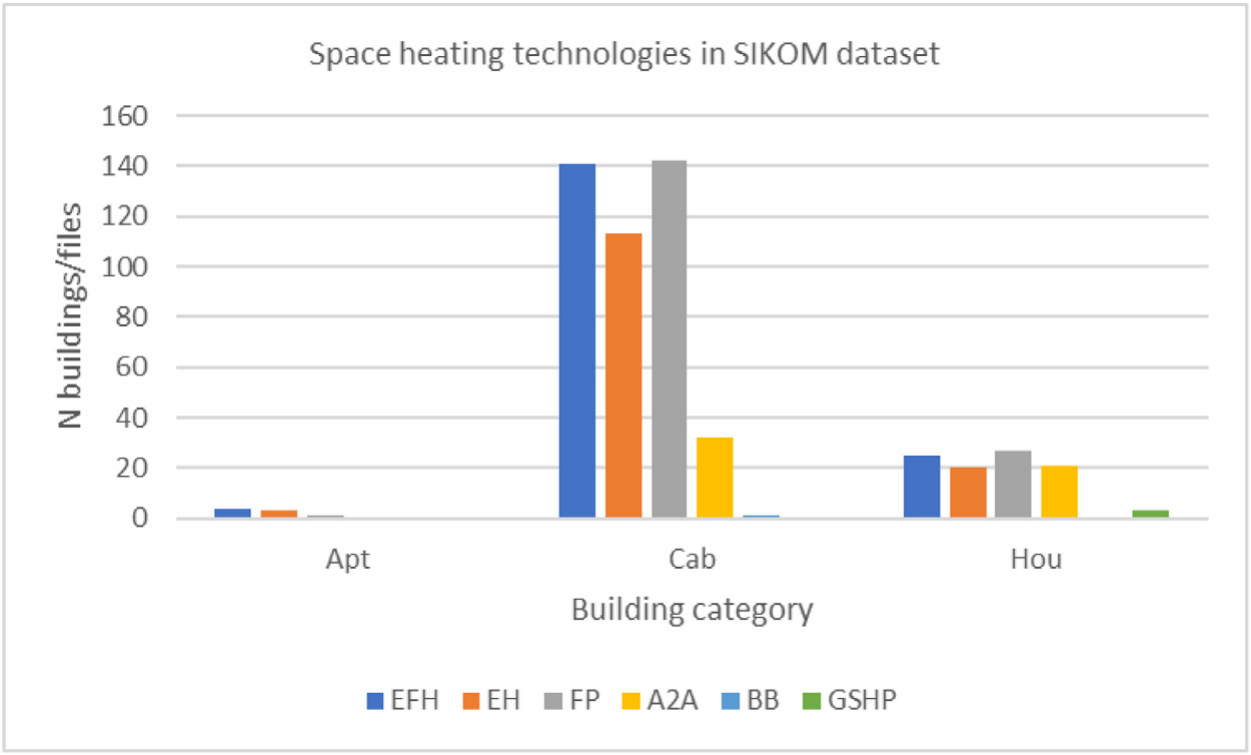
Space heating technologies given in the building information data for space heating the Sikom buildings in Dataset 3 per building category.
• Figure 6*: Given construction year for the units* split by building type.

**Fig. 6:**
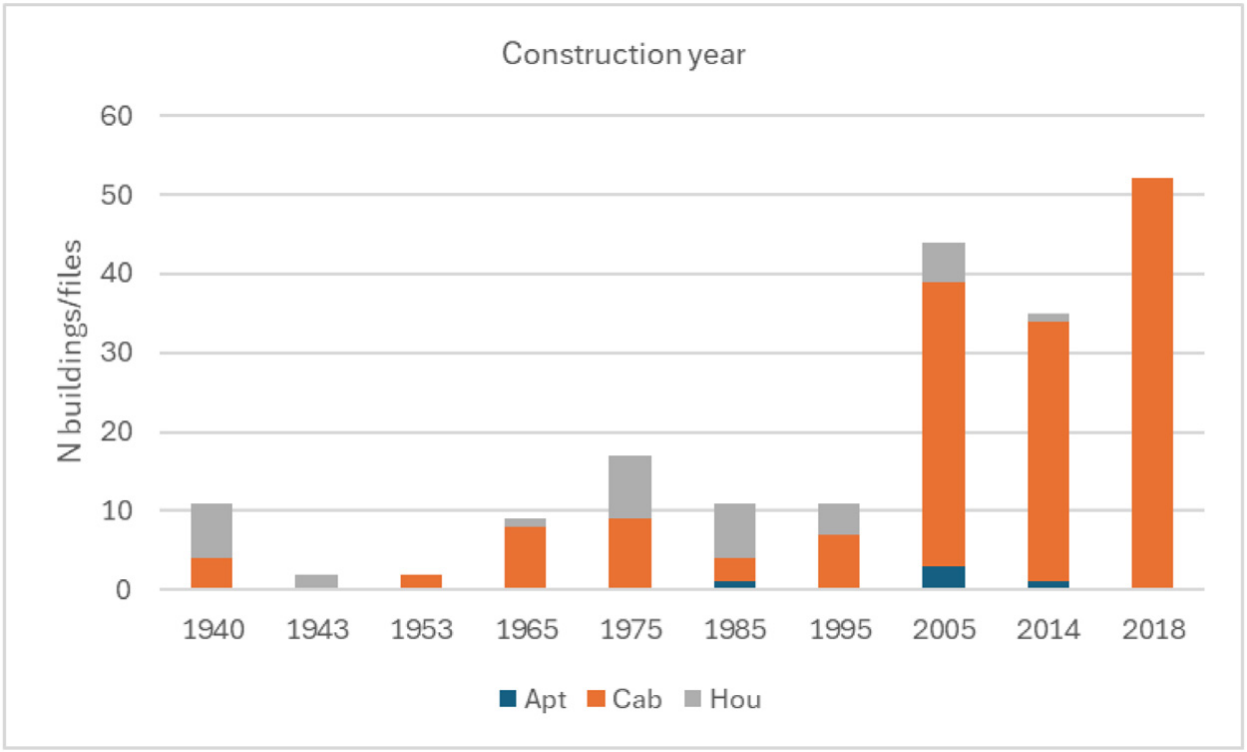
Construction year given in the building information data for the Sikom buildings in Dataset 3 per building category.
• Figure 7*: EV-charging information split per building type.*

**Fig. 7:**
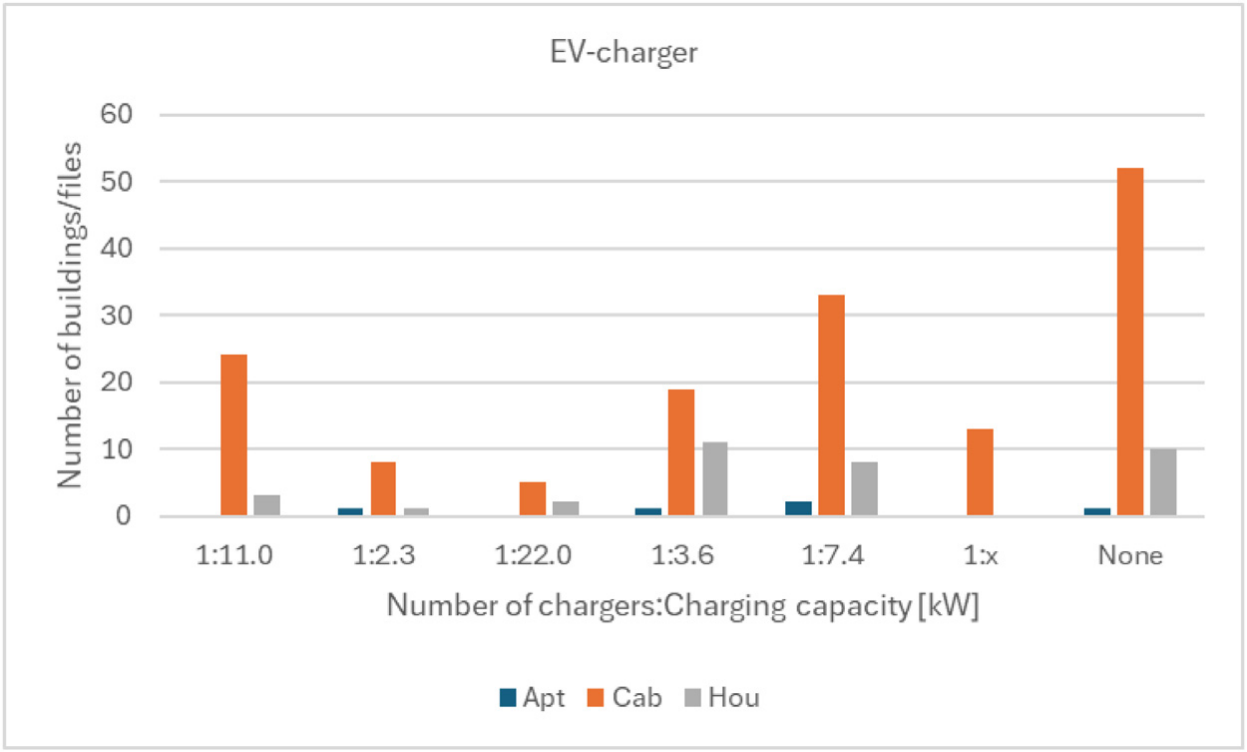
EV-charging information in the building information data for the Sikom buildings in Dataset 3 per building category.

While the files in dataset 3 contain electricity use data from several apartments, households and cabins, the costumers are not necessarily representative for Norwegian residential units. Given the building area statistics [18] one can assume that average apartments in Norway are approximately 70 m^2^ and average single-family houses are about 155 m^2^. New cabins are on average 99 m^2^ which is assumed to be bigger than the average cabin size of the cabin stock [19]. The buildings in Dataset 3 are on average bigger than the average residential units in Norway, as shown in Table 14. Fig. 6 also shows that most of the buildings are quite new compared to the general building stock of Norway, with most being built after 2005.

## Limitations

Users should exercise caution when down sampling the dataset, as missing data points may occur. Data from smart meters and district heating meters are generally reliable, as they comply with strict billing regulations. However, sub-meter data, while valuable, can vary in quality and should be validated before research use. In some cases, specific periods of sub-meter data may need to be discarded due to measurement errors [20].

For Dataset 1, the placement of heating energy meters means that *HtSpace* and *HtDHW* are derived from multiple meters, which introduces potential uncertainty. These measurements should therefore be interpreted with care.

The building information data in the files from Dataset 3 was collected from self-reports through user surveys and may contain inconsistencies due to differences in how respondents interpret the questions. The survey was conducted in Norwegian and the anonymized, non-linked responses and the full survey can be found in the data product for COFACTOR. Dataset 3 are not necessarily representative of the Norwegian cabin stock.

Overall, the datasets include mostly raw measurements, except for some exceptions described in the previous section, and each file should be inspected to confirm data quality and suitability for the intended analysis.

## Ethics Statement

The authors have read and follow the *ethical requirements* for publication in Data in Brief and confirming that the current work does not involve human subjects, animal experiments, or any data collected from social media platforms.

## Credit Author Statement

For this data descriptor: **Åse Lekang Sørensen**: Writing - Original Draft, Data Curation, Validation, Project administration, Writing Review and editing**, Synne Krekling Lien:** Data Curation, Writing - Original Draft, Writing - Review & Editing. **Harald Taxt Walnum:** Data Curation, Writing - Original Draft**,** Writing - Review & Editing.

For the related research article: **Synne Krekling Lien:** Conceptualization, Data curation, Formal analysis, Investigation, Methodology, Software, Validation, Visualization, Writing – original draft, Writing – review & editing. **Harald Taxt Walnum:** Conceptualization, Data curation, Formal analysis, Investigation, Methodology, Software, Validation, Visualization, Writing – original draft, Writing – review & editing. **Igor Sartori**: Conceptualization, Methodology, Validation, Writing – original draft, Writing – review & editing.

## Data Availability

Data.sintef.noCOFACTOR-Residential Dataset 1-3 (Original data)
